# Upregulation of sestrins protect atriums against oxidative damage and fibrosis in human and experimental atrial fibrillation

**DOI:** 10.1038/srep46307

**Published:** 2017-04-11

**Authors:** Zengxiang Dong, Chaolan Lin, Yujiao Liu, Hongbo Jin, Hong Wu, Zhenjun Li, Liping Sun, Lu Zhang, Xi Hu, Yingying Wei, Chengcheng Wang, Wei Han

**Affiliations:** 1 Department of Cardiology, The First Affiliated Hospital of Harbin Medical University, Harbin, China; 2 Intensive Care Unit, Sir Run Run Shaw Hospital, School of Medicine, Zhejiang University, Zhejiang, China; 3 Laboratory of Physiology, Harbin Medical University, Harbin, China; 4 Department of Cardiology, The Second Affiliated Hospital of Harbin Medical University, Harbin, China

## Abstract

Atrial Fibrillation (AF) is common in the elderly and Sestrins (Sesns) have been suggested to prevent age-related pathologies. The aim of this study was to investigate the effects of Sesns in AF. Clinical data were collected and a small sample of atrial appendage and atrium was obtained from patients undergoing valve repairment. The expression of Sesn1, Sesn2, and Sesn3 was significantly higher in patients with permanent atrial fibrillation (PmAF) than that in sinus rhythm (SR), and further greater in the left atrium than the right in PmAF patients. Superoxide anion and malondialdehyde were enhanced and positively correlated to the protein expression of Sesn1/2/3. Reactive oxygen species (ROS) production and Ca^2+^ overload were significantly decreased and cell survival was enhanced by overexpression of Sesns 1/2/3 in cultured HL-1 cells. Conversely, knockdown of Sesn1/2/3 resulted in significantly increased ROS and Ca^2+^ overload. In addition, the overexpression of Sesn1/2 significantly reduced the proliferation of fibroblasts, as well as decreased the protein expression of collagen and fibronectin1 in angiotensin II-stimulated cardiac fibroblasts. Our study demonstrated for the first time that Sesns expression is significantly up-regulated in AF, which therefore may protect hearts against oxidative damage and atrial fibrosis.

Atrial fibrillation (AF), the most common sustained arrhythmia, affects <1% of individuals 50 to 59 years of age and 11–18% of those ≥85 years of age^1^,^2^. The lifetime risk of AF is 22–26% in men and 22–23% in women by 80 years of age in the US and Europe^3^,^4^. Aging is a slow, progressive increase in oxidative stress resulting in the accumulation of oxidized and modified proteins^5^. AF self-perpetuates as a result of structural and electrophysiological remodeling of the atria, which is increased by oxidative stress. Oxidative stress is present in human and experimental AF models, and antioxidants attenuate atrial electrophysiological remodeling^6^,^7^,^8^. Oxidative stress stimulates Ca^2+^/calmodulin-dependent protein kinase II (CaMKII) to affect intracellular Ca^2+^ handling and contribute to AF^9^. Sestrins (Sesns) are cytoplasmic stress proteins that accumulate in cells exposed to stress, hypoxia, or DNA damage^5^,^10^. Sesns were previously thought to be antioxidants that inhibit the accumulation of reactive oxygen species (ROS) by maintaining peroxiredoxin activity^11^,^12^,^13^. Overexpression of Sesn1, Sesn2, and Sesn3 reduces ROS levels, whereas depletion of Sesn1 or Sesn2 by RNA interference in cultured cells or Sesn2 knockout mice increases ROS levels^12^,^13^,^14^. Sesns are also involved in alleviating oxidative stress by eliminating ROS^15^,^16^,^17^. In addition, any condition that leads to ROS accumulation may induce Sesns expression^10^. Therefore, the increased atrial ROS in AF may increase the expression of Sesns. The objective of this study was to investigate whether Sesns are induced in human AF and whether they can alleviate oxidative stress, enhance cell survival and reduce fibrosis in paced HL-1 cells and cardiac fibroblasts.

## Results

### Clinical Characteristics of Study Patients

The clinical characteristics of patients are presented in Table 1. No significant difference was identified between the groups in terms of age, sex, ratio of rheumatic heart disease, left ventricular end-diastolic diameter, and ejection fraction. However, the left atrium dimension in PmAF group was larger than that in SR group (*P* < 0.05), and the use of warfarin and digoxin was more common in PmAF group than in SR group. All other medications were similar between the two groups. The mean AF duration was 4.6 ± 3.4 years in the PmAF group.

### Upregulation of Sesns in PmAF Patients and Cells

**Figure 1:**
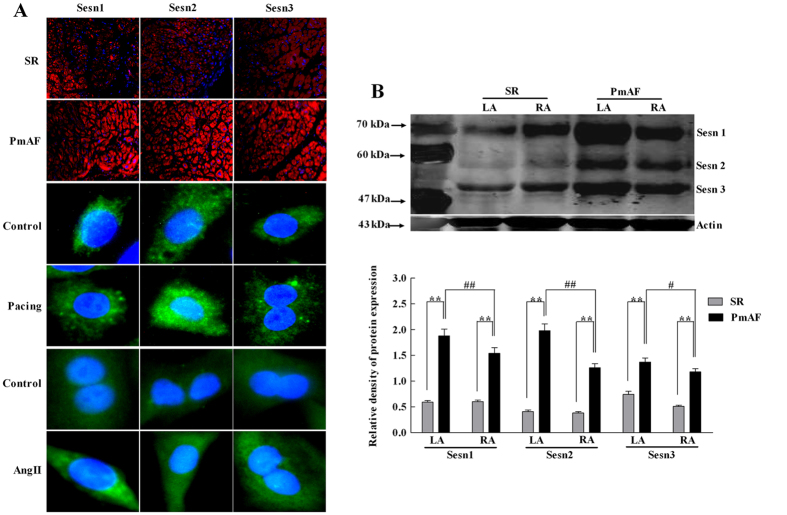
The expression of Sesns in atrium of patients and cells. (**A**) The location of Sesns proteins expressed in atria detected by immunofluorescence. Sesns proteins stained with red fluorescence. Nuclei stained with blue fluorescence. Magnification: ×100. The location of Sesns proteins expressed in HL-1 cells and cardiac fibroblasts detected by immunofluorescence. Sesns proteins stained with green fluorescence. Nuclei stained with blue fluorescence. Magnification: ×400. (**B**) The expression of Sesns protein detected with western blot. Lane1: Molecular weight standards; Lane 2: Left atrium in SR group; Lane 3: Right atrium in SR group; Lane 4: Left atrium in PmAF group; Lane 5: Right atrium in PmAF group. Quantification of relative immunoreactivity for bands, SR group n = 23, PmAF group n = 19 *P < 0.05, **P < 0.01 compared with the SR group; ^#^*P* < 0.05, ^##^*P* < 0.01 compared with the LA in the PmAF group. SR = Sinus rhythm; PmAF = Permanent AF; LA = left atrium, RA = right atrium.

The location of Sesns proteins in the atrium was detected with immunofluorescence. Red fluorescence, indicating the expression of Sesns proteins, was extensively seen in the cytoplasm and nucleus of cells in PmAF group. Sesn1, Sesn2, and Sesn3 were expressed in a larger quantity in PmAF than in SR (Fig. 1A). As shown in Fig. 1A, the expression of Sesn1, Sesn2, and Sesn3 also increased in the cytoplasm and nucleus of paced HL-1 cells and AngII- stimulated cardiac fibroblasts. Furthermore, Sesns expression by western blots confirmed that PmAF had significantly elevated expression of Sesn1 (*P* < 0.01), Sesn2 (*P* < 0.01), and Sesn3 (*P* < 0.01) (Fig. 1B). In the PmAF group, the expression of Sesn1 (*P* < 0.01), Sesn2 (*P* < 0.01), and Sesn3 (*P* < 0.05) in the LA was significantly higher than in the RA. Additionally, the basal expression level of Sesn 2 in SR patients is much lower than Sesn 1 (0.39 ± 0.13 vs. 0.60 ± 0.15, *P* < 0.01) and Sesn 3 (0.39 ± 0.13 vs. 0.63 ± 0.24, *P* < 0.01). However, Sesn2 sharply increased by more than four times in AF patients, whereas Sesn 1 and Sesn 3 did not change so much.

### Morphological Changes and the Relationship between Sesns and Oxidative Stress in Atrium of Patients

**Figure 2:**
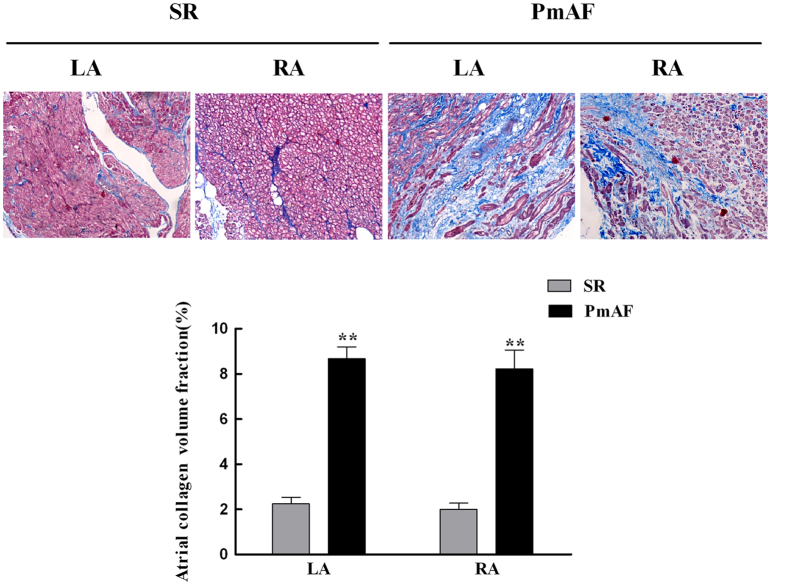
Histopathological changes in atrium of patients. Atrial collagen determined by Masson staining. Atrial collagen in the right and left atrium of patients with PmAF were significantly higher than in patients with SR. Quantification of atrial collagen is shown. SR group n = 23, PmAF group n = 19, **P < 0.01 compared with the SR group. SR = Sinus rhythm; PmAF = Permanent AF; LA = left atrium, RA = right atrium. Magnification: ×100.

**Figure 3:**
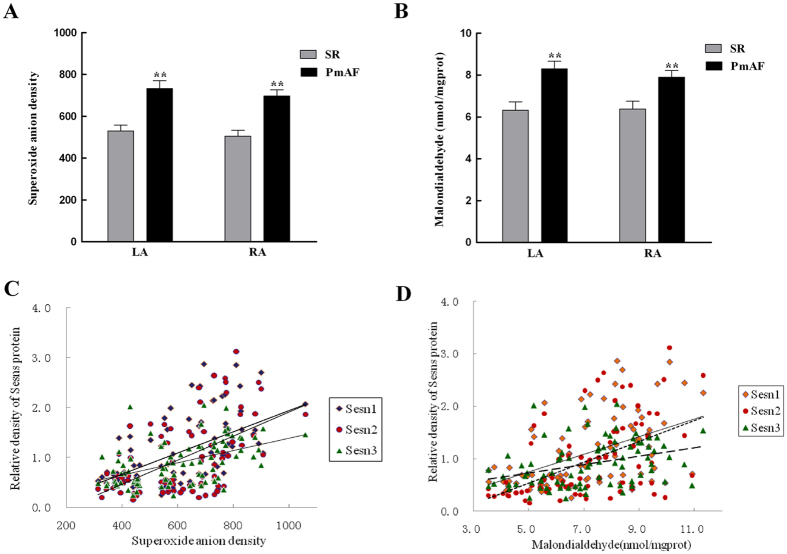
Relationship between oxidative stress and Sesns expression in patients. (**A**) The amount of O_2_·^−^ in SR and PmAF. (**B**) The amount of malondialdehyde in SR and PmAF. The production of O_2_·^−^and MDA from LA and RA increased significantly in PmAF group compared with that in SR group. (**C**) The amount of O_2_·^−^ positively correlated with the protein expression of Sesn1, Sesn2, and Sesn3. (**D**) A similar relationship was observed between MDA and Sesn1, Sesn2, and Sesn3. SR group n = 23, PmAF group n = 19, **P < 0.01 compared with the SR group. SR = Sinus rhythm; PmAF = Permanent AF; LA = left atrium, RA = right atrium.

Collagen detected by Masson staining showed that collagen volume fraction (CVF) was 2.24 ± 0.29% and 2.00 ± 0.27% in LA and RA of SR group, and this increased to 8.67 ± 0.53% and 8.22 ± 0.84% (all *P* < 0.01, vs. SR gruop) in LA and RA of AF group (Fig. 2). The amount of O_2_·^−^ production in atrium was higher in PmAF patients compared with that in SR patients (*P* < 0.01, Fig. 3A). Malondialdehyde accumulating from LA and RA also increased significantly in the PmAF group compared with that in SR group (*P* < 0.01, Fig. 3B). The amount of O_2_·^−^ was positively correlated to the protein expression of Sesn1 (r = 0.55, *P* < 0.01), Sesn2 (r = 0.57, *P* < 0.01), and Sesn3 (r = 0.49, *P* < 0.01) (Fig. 3C). A similar relationship was observed between MDA and Sesn1 (r = 0.48, *P* < 0.01), Sesn2 (r = 0.52, *P* < 0.01), and Sesn3 (r = 0.34, *P* < 0.01) (Fig. 3D).

### Sesns Inhibit Generation of ROS in Paced HL-1 Cells

**Figure 4:**
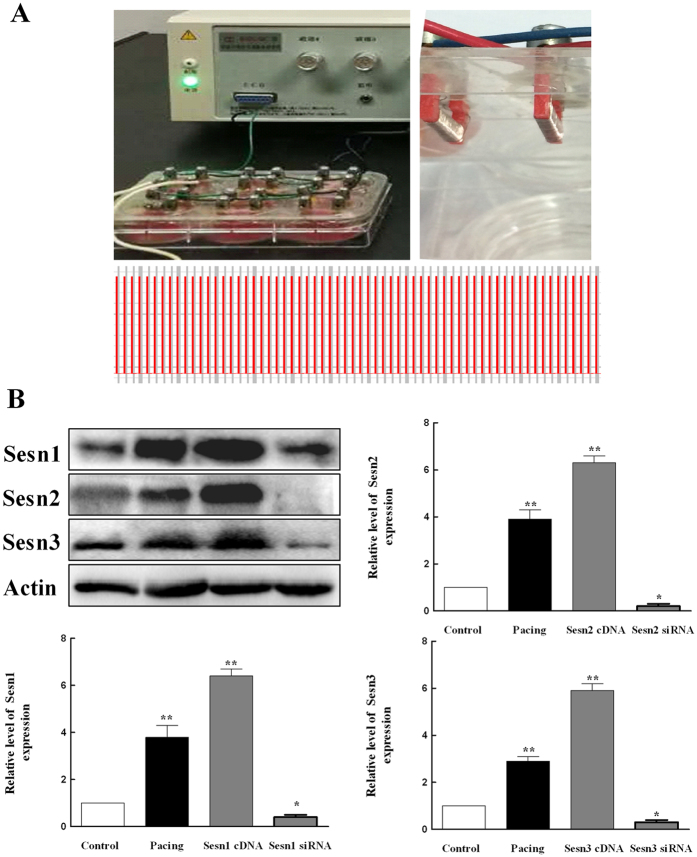
Field pacing and overexpression and knockdown of Sesns in HL-1 cells. (**A**) Left panel: YC-2-S stimulator and six-well plates equipped with electrode slices; Right panel: Low panel: Two paralleled platinum electrodes; Cells were stimulated in 600 times per minute. (**B**) The expression of Sesns protein detected with western blot. Representative lanes of immunoblotting. Lane1: Control; Lane 2: Pacing group; Lane 3: Overexpression of Sesns group; Lane 4: Knockdown of Sesns group. Quantification of relative immunoreactivity for bands, n = 6, **P < 0.01 compared with the control group.

**Figure 5:**
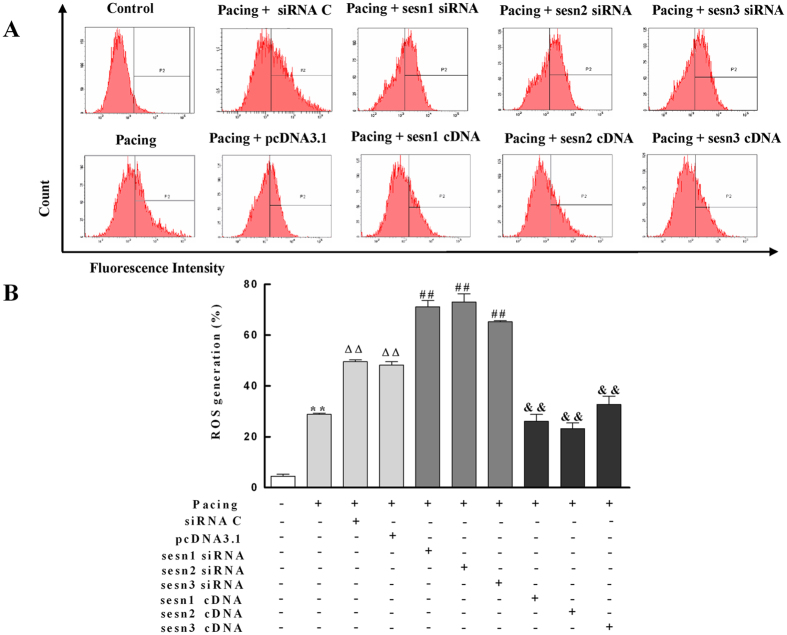
Effects of Sesns on ROS generation in paced HL-1 cells. (**A**) Flow cytometry was used to analyze the amount of ROS in paced HL-1 cells by transient transfection of Sesns siRNA and Sesns cDNA for 24 h. The transiently expressed Sesn1/2/3 siRNA increased the amount of ROS. The transiently expressed Sesn1/2/3 cDNA decreased the amount of ROS. (**B**) Quantification of relative ROS. n = 6, **P < 0.01 compared with the control group; ^ΔΔ^*P* < 0.01 compared with pacing group; ^##^*P* < 0.01 compared with the pacing + siRNA C group; ^&&^*P* < 0.01 compared with the pacing + pcDNA3.1 group.

Previous reports indicated that ROS played an important role in the development of AF^6^. To investigate the effects of Sesns on ROS in paced HL-1 cells, we expressed Sesns cDNA or Sesns siRNA in paced HL-1 cells (Fig. 4A). The overexpression and knockdown of Sesns were documented by western blots (Fig. 4B). The amount of ROS was analyzed by flow cytometry (Fig. 5A). Pacing increased the generation of ROS in HL-1cells from 4.5 ± 0.8% in control group to 28.8 ± 0.5% in paced group (*P* < 0.01), and Sesn1, Sesn2, and Sesn3 siRNA increased the amount of ROS to 71.1 ± 0.3%, 73.3 ± 3.3% and 65.3 ± 0.4%, respectively (Fig. 5B, *P* < 0.01). Moreover, the transiently expressed Sesn1, Sesn2, and Sesn3 cDNA decreased the amount of ROS to 26.1 ± 2.7%, 23.2 ± 2.3%, 32.7 ± 3.3%, respectively in paced HL-1cells (Fig. 5B, *P* < 0.01).

### Sesns Prevent Ca^2+^ Overload in Paced HL-1 Cells

**Figure 6:**
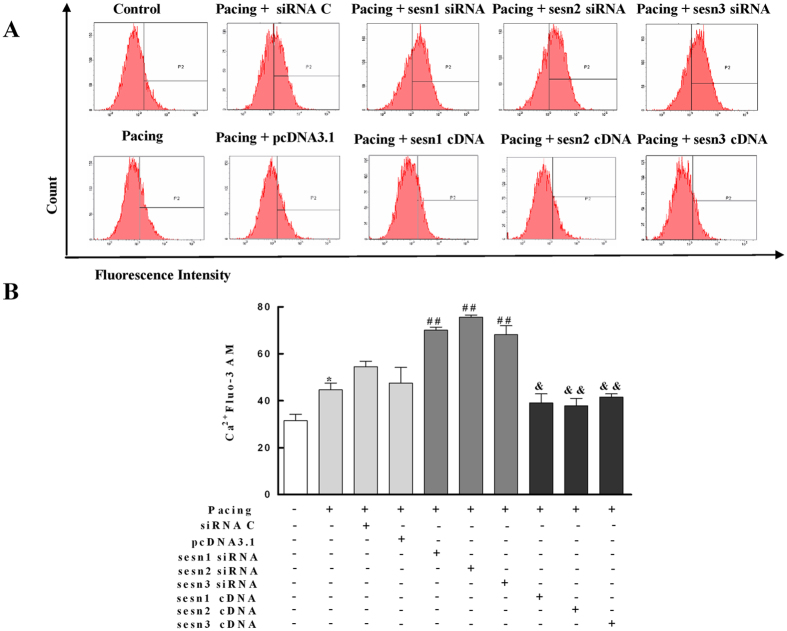
Effects of Sesns on Ca^2+^ in paced HL-1 cells. (**A**) Flow cytometry was used to analyze the amount of Ca^2+^ in paced HL-1 cells by transient transfection of Sesns siRNA and Sesns cDNA for 24 h. The transiently expressed Sesn1/2/3 siRNA increased the amount of Ca^2+^. The transiently expressed Sesn1/2/3 cDNA decreased the amount of Ca^2+^. (**B**) Quantification of relative Ca^2+^. n = 6, **P < 0.01 compared with the control group; ^##^*P* < 0.01 compared with the pacing + siRNA C group; ^&^*P* < 0.05, ^&&^*P* < 0.01 compared with the pacing + pcDNA3.1 group.

Intracellular Ca^2+^ was also monitored fluorescently in HL-1 cells. Figure 6 shows the Ca^2+^ concentration changes in HL-1 cells. Under controlled conditions, small variations in the Ca^2+^ concentrations were seen. Pacing significantly enhanced the Ca^2+^ concentration in HL-1 cells (*P* < 0.05). The transient transfection of Sesn1, Sesn2, and Sesn3 siRNA significantly increased the level of Ca^2+^ in paced HL-1 cells (*P* < 0.01). The elevation in intracellular Ca^2+^ was dramatically decreased by transiently expressing Sesn1, Sesn2, and Sesn3 cDNA in paced HL-1 cells (*P* < 0.01).

### Sesns Enhanced Survival of Paced HL-1 Cells

**Figure 7:**
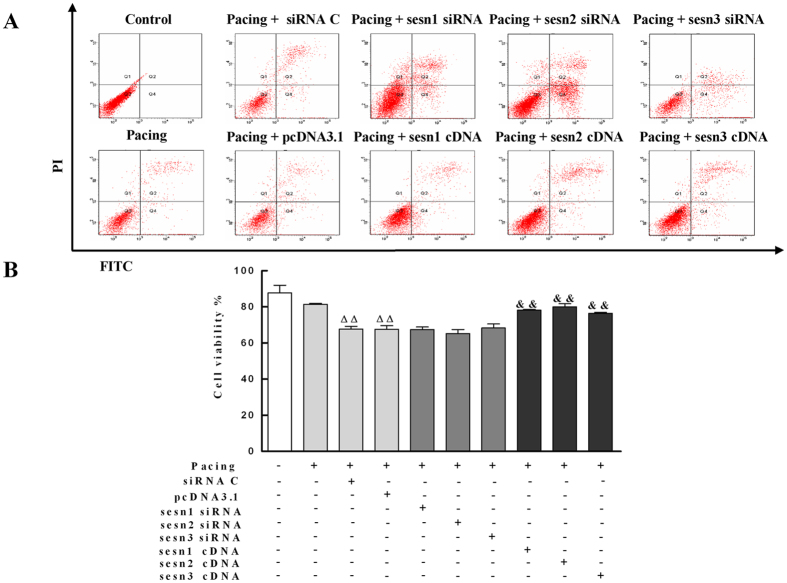
Effects of Sesns on survival rate of paced HL-1 cells. (**A**) The survival of HL-1 cells after transient transfection of Sesns siRNA and Sesns cDNA was monitored by flow cytometry. (**B**) Quantification of survival rate. Under control conditions, very small fluorescent variations were present in HL-1 cells. Pacing did not significantly change the survival rate of HL-1 cells. The silencing of Sesns by transient transfection of Sesn1/2/3 siRNA did not decrease the survival rate of paced HL-1 cells. The transiently expressed Sesn1/2/3 cDNA significantly enhanced the survival of paced HL-1 cells. n = 6, ^ΔΔ^*P* < 0.01 compared with pacing group; ^&&^*P* < 0.01 compared with the pacing + pcDNA3.1 group.

The survival rate of HL-1 cells was monitored by flow cytometry. Under controlled conditions, very small variations in the fluorescence were seen in HL-1 cells. Pacing together with transfection of PcDNA3.1 control significantly decreased the survival rate of HL-1 cells (67.5 ± 2.1% vs. 87.8 ± 4.1%, *P* < 0.01) and transiently expressed Sesn1, Sesn2, and Sesn3 cDNA significantly enhanced the survival of paced HL-1 cells form 67.5 ± 2.1% to 78.1 ± 0.5% (*P* < 0.01), 80.0 ± 1.7% (*P* < 0.01), 76.4 ± 0.5% (*P* < 0.01), respectively (Fig. 7). Unexpectedly, the knockdown of Sesns by transient transfection of Sesn1, Sesn2, and Sesn3 siRNA did not decrease the survival rate of paced HL-1 cells.

### Sesns Suppress AngII-induced Proliferation of Cardiac Fibroblasts and Collagen Synthesis

**Figure 8:**
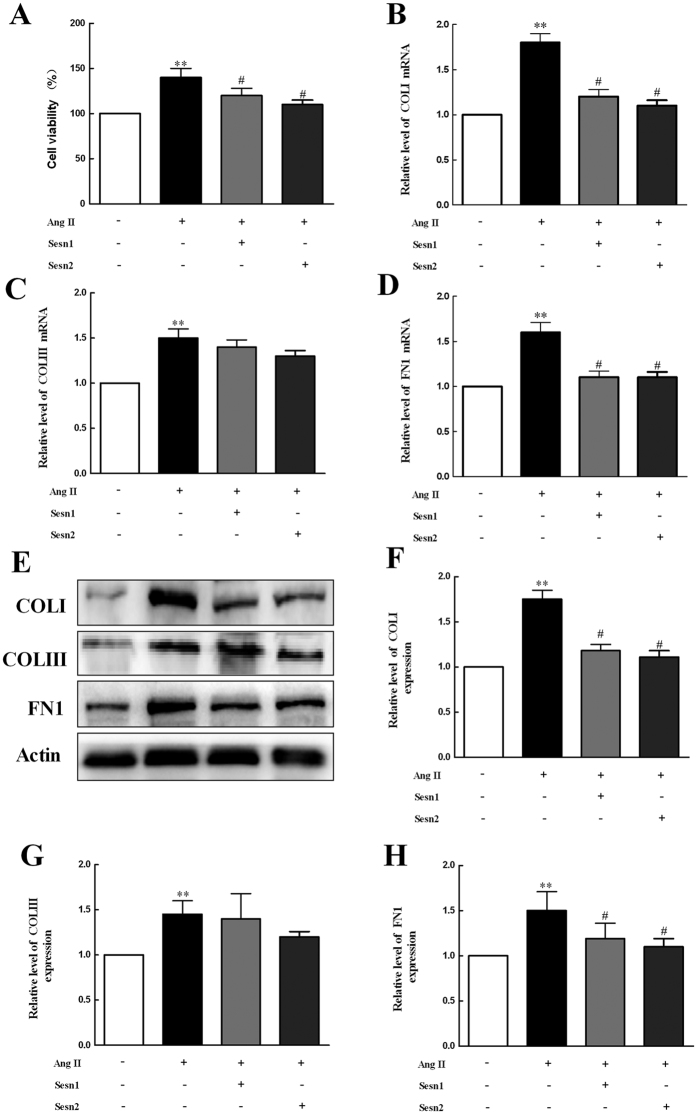
Sesns prevent AngII-induced proliferation of cardiac fibroblasts and collagen synthesis. (**A**) CCK8 method was used to analyze the proliferation of cardiac fibroblasts induced by AngII or/and transient transfection of Sesn1/2 cDNA. (**B**–**D**) mRNA levels of COLI, COLIII and FN1 in cardiac fibroblasts induced by AngII or/and transient transfection of Sesn1/2 cDNA were measured by RT-PCR. (**E**–**H**) Protein levels of COLI, COLIII and FN1 in cardiac fibroblasts induced by AngII or/and transient transfection of Sesn1/2 cDNA were measured by western blot. Data are expressed as mean ± SEM (n = 6). **P < 0.01 compared with control group; ^#^*P* < 0.05 compared with Ang II group.

To study the effect of Sesns on cardiac fibrosis, we used CCK-8 method to detect the proliferation of cardiac fibroblasts (CFs). As Fig. 8A shows that the overexpression of Sesn1 and Sesn2 in cardiac fibroblasts significantly reduced the proliferation of fibroblasts (*P* < 0.05). Next, we analyzed mRNA expression levels for COL I/III and FN1. As shown in Fig. 8B–D, Ang II induced increases of COL I/III and FN1 mRNA expression (*P* < 0.01). In contrast, increased the expression of Sesn1 and Sesn2 significantly attenuated the Ang II-induced COL I and FN1 mRNA expression (*P* < 0.05). To validate the mRNA findings, we identified the effect of Sesn1 and Sesn2 on protein expression of COL I/III and FN1. As shown in Fig. 8E–H, COL I/III and FN1 were severely increased induced by AngII (*P* < 0.05) and the effect were clearly inhibited by Sesn1/2-overexpressing (*P* < 0.05). These results demonstrated Sesn1 and Sesn2 negatively regulated AngII-mediated CFs proliferation.

## Discussion

### Main Findings of the Study

The present study demonstrated for the first time that Sesns expression in RA and LA is significantly up-regulated in PmAF patients compared with that in SR. Higher O_2_·^−^ and MDA are associated with higher Sesns expression. Furthermore, the protective effects of Sesns were confirmed by gene overexpression and knockdown in paced HL-1 cells and cardiac fibroblasts. Sesns overexpression significantly decreased oxidative stress and enhanced cell survival in paced HL-1 cells, whereas Sesns knockdown with siRNA induced ROS accumulation. Additionally, overexpression of Sesn1 and Sesn2 inhibited proliferation and collagen synthesis in cardiac fibroblasts stimulated by Ang II. Therefore, induced as an endogenous protective factor, Sesns, may protect against oxidative damage and fibrosis in AF.

### Comparison with Previous Studies

Sesns are cytoplasmic stress proteins that accumulate in cells exposed to stress, hypoxia, and DNA damage^5^,^10^. Here, we identified significantly higher levels of Sesns are expressed in patients with PmAF than SR, which may be attributed to an increased oxidative stress in PmAF patients (Fig. 3). The same increase was also seen in paced HL-1 cells (Fig. 5). Studies have demonstrated that AF itself induces substantial ROS and oxidative stress in fibrillating atrial tissue^18^,^19^, thus may induce Sesns upregulation in such condition. These results are consistent with a previous study which showed that any condition which leads to ROS accumulation may induce Sesns expression^10^,^20^. Interestingly, the expression of Sesns was higher in LA than RA in PmAF, which may partly due to AF cause greater changes in O_2_·^−^ production in the LA or LAA than in the RA or RAA^18^. Also, there was a trend in the more increase of oxidative stress in LA than RA in PmAF suggested by of MDA in our study, although no statistical difference were observed (P = 0.07). An animal study of atrial tissue in AF shows a gradient of oxidative stress with greater oxidative stress on the left^18^, however, it is not the case in human^21^,^22^. Thus, besides oxidative stress, other mechanisms such as pressure, endocrine function and Ca^2+^ homeostasis^19^,^23^ of LA, by which the LA is distinguished from the RA, should be considered and require further investigation.

Noticeably, our study shows that there are significant basal expression level differences in Sesns. The expression of Sesn 2 in SR patients is much lower than Sesn 1 and Sesn 3, but increase more sharply than Sesn 1 and Sesn 3 in AF. This would suggest that Sesn 2 is the main subtype induced in oxidative stress in AF. Similarly, the same trend was noticed in the paced HL-1 cells in this study. Evidences were also shown in other studies that Sesn 2 is the main stress proteins induced in oxidative damage and provide significant protection in cardiovascular diseases^24^,^25^. Therefore, different potencies of Sesns are induced in AF, which may suggest Sesn 2 play more important role in the pathological process of AF, whereas Sesn 1 and Sesn 3 work both in physiological and pathological conditions.

Oxidative stress drives electrical and structural remodeling, which contributes to the development and progression of AF. Anti-inflammatory and antioxidant agents prevent atrial electrical remodeling in animal models of AF and reduce the incidence of postoperative AF in humans^6^,^7^,^8^. Heat shock proteins (HSPs), cytoplasmic stress proteins, are increased in AF and protect against atrial tachycardia-induced remodeling in cellular and animal models^26^,^27^,^28^. Treatment with geranylgeranylacetone (GGA), an HSP inducer, increases HSP expression, suppresses refractoriness, and prevents AF in dogs subjected to atrial tachypacing^26^. However, data are conflicting. The previous study suggested that GGA alone, without ischaemia, does not alter electrical conduction or AF duration^29^. Although the expression of Hsp 27 is significantly increased in paroxysmal AF compared with SR and persistent AF, no changes are observed in the expression of most HSPs, such as Hsp40, Hsc70, Hsp70, and Hsp90^28^. Like HSP, Sesns are also cytoplasmic stress proteins, which exhibit oxidoreductase activity and may function as antioxidants^12^. However, Sesns are widely expressed even in the absence of exogenous stress. In drosophila, expression of dSesn is increased upon maturation^10^,^20^. In humans, Sesn1, Sesn2, and Sesn3 are all expressed in atria without stress (shown in Fig. 1). Besides physiological conditions, we show for the first time that all kinds of Sesns are upregulated in human AF or paced HL-1 cells. These results have been consistent with previous studies, which show Sesns are cytoplasmic stress proteins accumulating in cells exposed to oxidative stress^5^,^12^. Here Sesns also display protection against oxidative damage and fibrosis in experimental AF, conforming to studies that Sesns has potential to limit liver damage and fibrosis in chronic ER stress and protect renal tubules again stress during acute kidney injury^30^,^31^.

### Sesns as a Protective Factor

The physiological functions of Sesns remain poorly defined. To our knowledge, this is the first study to report that all Sesns upregulate in human AF and protect paced cardiac myocytes against oxidative stress. Here we show that Sesns are positively correlated with oxidative stress in humans and paced HL-1 cells, which suggested oxidative stress may be the cause of Sesns upregulation in such condition. As a results, Sesns compensatory upregulate to antagonize injury induced by oxidative stress. However, this kind of compensatory mechanism is not enough to prevent the oxidative stress in AF, considering the fact that ROS accumulates in human AF and paced atrial cells in spite of theirs upregulation. Furthermore, the overexpression of Sesns decreases the amount of ROS, enhances cell survival and inhibits fibrosis in paced HL-1 cells and cardiac fibroblasts, suggesting that exogenetic Sesns may be protective and useful in AF. Some other studies also suggest that Sesns may play an important role in the regulation of cardiac pathophysiology and provide protection^24^,^32^,^33^. In Drosophila, heart-specific depletion of dSesn caused heart dilation, arrhythmias, and malfunction^10^. Sesn2 proteins were found to accumulate in the heart during ischemic conditions protecting the heart against ischemia and reperfusion injury^24^. Sesn1 inhibits angiotensin II-induced fibroblast proliferation and collagen production in cardiac cells^34^. Thus, the increase of Sesns in AF patients supports the notion that Sesns induced by ROS are an endogenous compensatory mechanism to prevent AF developing.

The mechanisms by which various types of Sesns provide cardioprotection in AF are not fully understood. The protective function of Sesns in AF is probably mediated by two pathways. In one pathway, the antioxidant activity of Sesns *in vitro* and *in vivo* alleviates the oxidative damage and protects in AF^10^,^11^,^12^,^13^,^14^. In the present study, Sesns overexpression decreased ROS production by 20–30% in paced HL-1 cells. In the second pathway, Sesns inhibit fibrosis, which is an important structural contributor to formation of AF substrate. We show that Sesn1 and Sesn2 inhibit cell proliferation and fibrosis in cardiac fibroblasts stimulated by AngII, which is in accordance with the findings of another study^34^. Target of rapamycin (TOR) and TGF-beta signals are two possible mechanisms associated in the anti-fibrosis function of Sesns^35^,^36^,^37^. Hyperactivity of TOR is related to activation of cardiac fibroblasts in stress^35^, and the inhibition of TOR can ameliorate the chronic pressure-induced left ventricular hypertrophy and cardiac fibrosis^36^. As an inhibitor of TOR signaling^10^,^11^, Sesns thereby presumably attenuate fibrosis in AF.

However, the protective function of Sesns may be independent of oxidative stress, since our data show the survival of paced HL-1 cells did not grow worse in Sesns knockdown with siRNA despite the fact that apparent effects of introducing Sesns on oxidative stress and survival. The previous study suggested that ROS can also be protective as signal preconditioning protection and induce stress responses that lead to survival^38^. Other mechanisms for protection are implied in the present study and should be further investigated in the future.

### Potential Significance of Sesns Upregulation in Atrial Fibrillation

Sesns are induced in patients with PmAF, which may serve as an endogenous protective mechanism to prevent AF. Oxidative stress has been implicated in the structural and electrophysiological remodeling of AF and antioxidants prevent atrial remodeling and AF incidence^7^,^8^. Thus, as antioxidants, Sesns probably attenuate the AF substrate and inhibit the remodeling of atria in AF^11^,^12^,^13^. Here we show the overexpression of Sesns decreases the amount of ROS, enhances cell survival in paced HL-1 cells, as well as inhibits proliferation and collagen production in cardiac fibroblasts induced by AngII. Sesns have been demonstrated to prevent age-related pathologies, such as cancer and type II diabetes^10^,^32^,^39^. AF is also an age-related disease and, therefore, may be prevented by Sesns. Thus, just like the effects of B-type natriuretic peptide in heart failure^40^, our results provide novel evidences that Sesns are induced and provide protection in AF, and may serve as a possible therapeutic target in the clinical management of AF.

### Potential Limitations

First, we demonstrated the protection of Sesns in HL-1 myocytes and cardiac fibroblasts, but not directly in AF patients. Although HL-1 cells beat spontaneously, their electrophysiological, functional, and metabolic properties are modified during culture. Second, most of the patients included in the present study had rheumatic heart disease. Although the groups were well matched for rheumatic heart disease, rheumatic fever influences oxidative stress and therefore may affect the Sesns level. Third, we did not establish a cause and effect relationship between ROS and Sesns, as well as clarify if the actions of Sesns are direct or indirect on oxidative stress. Fourth, transfection with empty vector or control RNA causes a significant increase in oxidative stress, which would influence the results of the study. Another means to introduce nucleotides which could not affect oxidative stress should be used. Fifth, the contradiction between ROS and survival indicating extra mechanisms of cardioprotection by Sesns, which is independent of oxidative stress, are implied in the present study and should be further investigated in the future.

## Summary

Sesns are induced as an endogenous protective factor in AF, which protects the atria against oxidative damage and fibrosis. However, the compensatory increase of Sesns in AF is not enough to prevent this kind of disease, suggesting extra exogenetic Sesns are necessary and helpful for treatment of AF. This identification provides insight into the molecular basis of AF and suggests therapeutic targets for the common rhythm disturbances.

## Materials and Methods

### Patients

Tissue samples from the right atrium (RA) and left atrium (LA) appendage were obtained from 42 patients undergoing cardiac surgery for valvular replacement at the First Affiliated Hospital, and the Second Affiliated Hospital, Harbin Medical University, between 2011 and 2013. All patients had valvular heart disease and that a large number of patients had rheumatic heart disease. Patients were divided into two groups: permanent AF group (PmAF, n = 19) in which patients has an AF history of >6 months and electrocardiographically documented AF at the time of surgery; and sinus rhythm group (SR, n = 23) in which patients had no documented AF and no AF history. All patients were excluded liver and renal function abnormality, neoplastic disease and inflammation before surgery. The size and function of hearts were measured by echocardiography (Philips iE33, Holland). One part of the atrium and appendage tissue was fixed in 4% formalin for histopathological examination, and the remaining tissue was frozen in liquid nitrogen and stored at −80 °C for immunofluorescence and western blot and other analyses. The study was approved by the Ethics Committee of the First Clinical College of Harbin Medical University, and all patients gave informed consent. All the methods were carried out in accordance with the approved guidelines.

### HL-1 Cells Culture and Pacing

HL-1 atrial myocytes, derived from the adult mouse atria, were donated by Professor William Claycomb (Louisiana State University, New Orleans, LA, USA). The cells were cultured in complete Claycomb medium (JRH Biosciences, UK) supplemented with 100 μmol/L norepinephrine (consisting of 10 mmol/L norephinephrine (Sigma, USA) dissolved in 0.3 mmol/L L-ascorbic acid (Sigma, USA)), 4 mmol/L L-glutamine (Gibco, The Netherlands), and 10% FBS (Gibco, The Netherlands). The myocytes were incubated in flasks coated with 0.02% gelatin (Sigma, USA) and 12.5 μg/mL fibronectin (Sigma, USA) at 37 °C in a 5% CO_2_ atmosphere. The basal frequency of the HL-1 cells was about 0.5 Hz. Cell models of AF were established by field stimulation 600 times per minute for 12 h (10 Hz/5 ms, 1.0 V/cm; YC-2-S stimulator, Chengdu Instrument Factory, China). Two platinum electrodes, which were positioned parallelly were used in the field pacing of HL-1 cells (shown in Fig. 4).

### Cardiac Fibroblasts Isolation and Culture

Cardiac fibroblasts were isolated from neonatal SD rats (from the animal laboratory center of the Second Affiliated hospital of Harbin Medical University). All animal experimental procedures were approved by the ethical committee of Harbin Medical University, China and conform to published NIH guidelines for animal research (NIH Publication No. N01-OD-4–2139, 8^th^ edition, 2011). Hearts were cut into pieces and 0.25% trypson-EDTA Solution (Beyotime biotechnology, China) was added to cells for 20–30 times. Digestive fluids were matched up one-to-one with HG-DMEM (high glucose Dulbecco’s modified Eagle medium, Gibco, The Netherlands) supplemented with 10% fetal bovine serum (FBS, Gibco) and 1% 1X Penicillin-Streptomycin Solution (Gibco, The Netherlands). Then the cells were filtered and centrifuged at 2000 rpm, 4 °C for 5 minutes. Cardiomyocytes were isolated from adherent fibroblasts after incubation for 1.5 h. 0.25% trypson-EDTA and Phenol Red (Gibco, The Netherlands) was used in fibroblasts passage. The fibroblasts could be harvested while 2 times of the passages. All cells were incubated with above culture at 37 °C, 5% CO_2_. 1 μM Ang II was used to stimulate cardiac fibroblasts. The proliferation of the cells was measured by CCK-8 assay.

### Sesns Overexpression and Knockdown

Total RNA was extracted from HL-1 cells using RNAiso Plus (Takara D9108A) and cDNA was obtained using reverse transcription. Primers were synthesized by Harbin Boshi Biotechnology limited company (shown in Supplement data 1). The PCR reaction mixture included: cDNA template 2.5 μl; Ex Taq 0.5 μl; dNTP 5 μl; 10 × PCR Buffer 5 μl; Sensns 2 μl; antisense 2 μl; and dd H_2_O 33 μl. Thirty-five cycles of PCR amplification were performed as follows: 94 °C for 10 minutes, 94 °C for 30 seconds, 60 °C for 30 seconds, 72 °C for 2 minutes, and 72 °C for 10 minutes. PCR products were purified with 1% agarose gel electrophoresis using AxyPrep DNA purification kit (Axygen, USA). PcDNA3.1-Sesn1, PcDNA3.1-Sesn2, and PcDNA3.1-Sesn3 were constructed by cloning cDNA into the EcoR I/Kapn I site of pcDNA3.1 vectors (Invitrogen, USA). Short interfering RNAs targeting Sesn1, Sesn2, Sesn3, and negative control were synthetized by GenePharma co, Ltd (Shanghai, China) (shown in Supplement data 2). HL-1 cells were transfected with PcDNA3.1-Sesns for 72 h or siRNA targeting Sesns for 72 h by Lipofectamine 2000 (Life Technologies, USA) in 6-well plates. Similarly, Sesn1 and Sesn2 were overexpressed in cardiac fibroblasts. Overexpression and silencing of Sesns were determined by SYBR real-time quantitative PCR with One Step SYBR PrimeScript RT-PCR Kit II (TaKaRa) (Primer sequences were shown in Supplement data 3), as well as by western blots.

### Immunofluorescence

Immunofluorescence staining for Sesn1, Sesn2, and Sesn3 was performed on frozen sections. Slides were permeabilized with 0.4% Triton X-100 in phosphate-buffered saline (PBS), washed, and blocked with 10% bovine serum albumin (BSA) at room temperature. The sections were incubated with anti-Sesn1 (Abcam, USA), anti-Sesn2 (Santa Cruz Biotechnology, USA), and anti-Sesn3 (Abcam, USA) antibodies, overnight at 4 °C. Slides were incubated with Alexa Fluor 594 antibodies (Molecular Probes, USA) at room temperature. Nuclei were stained with 4′, 6-diamidino-2-phenylindole (DAPI, Sigma-Aldrich, USA) and images were captured by a laser-scanning confocal microscope (Olympus, Japan). Three paraffin sections were made for every patient and three fields were evaluated in every section. Cells were seeded on glass coverslips and cultured for 24 h in DMEM (10% FBS). Cells were fixed with 4% paraformaldehyde for 20 min at room temperature. Cells were washed with PBS and blocked with 3% BAS for 1 h, then incubated with anti-Sesn1 (Abcam, USA), anti-Sesn2 (Santa Cruz Biotechnology, USA), and anti-Sesn3 (Abcam, USA) antibodies, overnight at 4 °C. They were incubated with secondary antibody (FITC, EarthOx, USA) for 2 h at 37 °C. Nuclear staining was incubated with DAPI (Sigma, St. Louis, MO, USA) at room temperature for 10 min. Cells were imaged with Carl Zeiss Axio VertA1 microscope (Carl Zeiss Microimaging, Thornwood, NY, USA).

### Real-time Quantitative Polymerase Chain Reaction (qRT-PCR) Analysis

A quantitative analysis of collagen I (COLI), collagen III (COLIII) and fibronectin1 (FN1) mRNA expression in cardiac fibroblasts was determined by qRT-PCR. Total RNA was extracted from cardiac fibroblasts using Trizol reagent (Invitrogen, USA) according to manufacturer’s protocols. 0.5 μg of total RNA was reverse transcribed with High-Capacity cDNA Reverse Transcription Kit (Applied Biosystems, USA) to obtain cDNA. The SYBR Green PCR Master Mix Kit (Applied Biosystems, USA) was used in qRT-PCR to quantify the RNA levels of COL I, COL III and FN1 in cardiac fibroblasts, with GAPDH as an internal control. The qRT-PCR was performed on 7500 FAST Real-Time PCR System (Applied Biosystems, USA) for 40 cycles. The sequences of primers used for amplification were: COLI, 5′-CGTGGAAACCTGATGTATG CT-3′ and 5′-CCTATGACTTCTGCGTCTGG-3′; COLIII, 5′-GATCCTAACCAAGGCTGC AA-3′ and 5′-ATCTGTCCACCAGTGCTTCC-3′; FN1, 5′-GACACTATGCGGGTCACT TG-3′ and 5′-CCCAGGCAGGAGATTTGTTA-3′; GAPDH, 5′-AAGAATGGTGAAGCAG GC-3′ and 5′- TCCACCACCAGTTGCTGTA-3′.

### Western Blot Analysis

The protein concentration in the supernatant was determined by BCA assay. Aliquots of 80 μg protein were separated in SDS-PAGE and transferred onto nitrocellulose membranes by an electric transfer (BIORAD Inc., USA). The membranes were blocked with 5% milk PBS-Tween20 (PBST) and incubated with primary antibody including: anti-Sesn1 (Abcam, USA), anti-Sesn2 (Santa Cruz Biotechnology, USA), anti-Sesn3 (Abcam, USA), anti-COL I (Abcam, USA), anti-COL III (Abcam, USA), anti-FN1 (Abcam, USA), and anti-Actin (Cell Signaling, USA) antibodies. They were then incubated with Alexa Fluor 700 antibodies (Molecular Probes, USA). Images were captured on an Odyssey Infrared Imaging System (LI-COR Biosciences, USA), and quantified using Odyssey v1.2.

### Histopathological Examination

Masson’s trichrome staining for interstitial collagen deposition were analysed. Tissue specimens were fixed in 4% paraformaldehyde and embedded in paraffin. Serial sections about 5 μm in thickness were cut and stained with Masson trichrome. Sections were photographed with an Olympus HPISA-1000 camera and the extent of fibrosis was quantified with Image-Pro Plus 6.0 (Media Cybernetics, Bethesda, Md). Sections of the heart were analysed for bright blue staining (collagen) and red staining (cardiacmyocyte). Collagen volume fraction (CVF) was defined as the sum of all stained interstitial collagen tissue areas divided by the whole tissue area.

### Assessment of Oxidative Stress Markers

The amount of superoxide anion (O_2_·^−^) in the atrium was measured as described previously^18^. Electron spin resonance spectroscopy was used to examine intracellular O_2_·^−^ production with the cell-permeable spin probe 1-hydroxy-3-methoxycarbonyl-2, 2, 5, 5- tetramethylpyrrolidine hydrochloride (CMH; Alexis Corp). Briefly, freshly isolated atrial tissues was incubated with deferoxamine-chelated Krebs-HEPES solution containing CMH (0.5 mmol/L), deferoxamine (25 μmol/L), and DETC (5 μmol/L) for 90 minutes at 37 °C. Then, samples were transferred into 1-mL syringes filled with Krebs-HEPES solution and frozen in liquid nitrogen. Samples were scanned with a Bruker EMX spectrometer. Analyses of the spectra peak height were used to quantify the amount of O_2_·^−^ produced by the tissue. The amount of malondialdehyde (MDA) produced from the atrium was examined by thiobarbituric acid (TBA) assay with an MDA Detection Kit (Nanjing Jiancheng Bioengineering Institute, China). In brief, stored atrium samples were weighed and 5% tissue homogenate was obtained on ice in isotonic Na chloride. 0.1 ml tissue homogenate was processed according to the manufacturer’s instructions and detected spectrophotometrically at 532 nm by an ultraviolet spectrophotometer (GeneQuant pro, Amersham Biosciences, England).

### Measurement ROS, Ca^2+^ and Apoptosis

HL-1 cells were treated with different group and incubated with 10 μM DCFH-DA (Sigma-Aldrich, USA) at 37 °C for 30 minutes for ROS detection. Fluorescence intensities of DCF were measured by excitation wave length of 488 nm and emission wave length of 525 nm with flow cytometry (BD FACSCantoII). HL-1 cells were also loaded with the Ca^2+^ indicator Fluo 3/AM (5 μM) (Beyotime Biotechnology, China) at 37 °C for 30 minutes to measure the Ca^2+^ content within HL-1 cells. Fluo 3/AM excitation wavelength was 488 nm and emission wavelength was 525 nm. Surviving cells of different treatment group were tracked with flow cytometry. Cell samples were prepared according to manufacturer’s instructions of Apoptosis Detection Kit I (BD Sciences, USA). The data of flow cytometry was acquired using Cell Quest software.

### Statistical Analysis

Quantitative data were expressed as mean ± SEM. Differences among quantitative data were analyzed by ANOVA. Multiple comparisons were made using SNK-q test. An unpaired student’s t-test was used for comparisons between two groups. Categorical variables were analyzed by Chi-square test with continuity correction when 1 ≤ T < 5. Pearson’s correlation coefficient (r) was used to measure the strength of association between quantitative parameters. A 2-tailed *P* < 0.05 was considered statistically significant.

## Additional Information

**How to cite this article**: Dong, Z. *et al*. Upregulation of sestrins protect atriums against oxidative damage and fibrosis in human and experimental atrial fibrillation. *Sci. Rep.* **7**, 46307; doi: 10.1038/srep46307 (2017).

**Publisher's note:** Springer Nature remains neutral with regard to jurisdictional claims in published maps and institutional affiliations.

## Supplementary Material

Supplementary Material

## Figures and Tables

**Table 1:** Clinical characteristics of study patients.

	SR (n = 23)	PmAF(n = 19)
Age, y (mean ± SD)	54.2 ± 8.5	56.3 ± 7.4
Sex, men/women	9/14	10/9
Duration of AF, y (mean ± SD)	0	4.6 ± 3.4 *
Rheumatic heart disease	8 (35%)	11 (58%)
Valvular heart disease		
Mitral valves involved	6 (26%)	7 (37%)
Aortic valves involved	8 (35%)	2 (11%)
Combined valvular disease	9 (39%)	10 (53%)
Diabetes mellitus	5 (22%)	1 (5%)
Hypertension	8 (35%)	3 (16%)
CHD	7 (30%)	2 (11%)
LA dimension, mm (mean ± SD)	37.6 ± 4.1	52.9 ± 9.8*
LV end-diastolic diameter, mm (mean ± SD)	45.7 ± 5.1	49.9 ± 5.6
LV ejection fraction, % (mean ± SD)	58.9 ± 4.9	55.1 ± 9.5
Aspirin	6 (26%)	9 (47%)
Warfarin	0	5 (26%)*
β-blockers	8 (35%)	10 (52%)
Digoxin	0	6 (32%)*
Amiodarone	0	1 (5%)
ACE inhibitors	3 (13%)	6 (32%)
Nitrates	2 (9%)	4 (21%)
Statins	2 (9%)	3 (16%)

SR = Sinus rhythm; PmAF = Permanent AF; LA = left atrium; LV = left ventricle; ACE = angiotensin-converting enzyme; ARB = angiotensin receptor blockers. *P < 0.05 compared with SR group.
