# Supplementary material for: Upregulation of sestrins protect atriums against oxidative damage and fibrosis in human and experimental atrial fibrillation
Source: Sci Rep. 2017 Apr 11;7:46307. doi: 10.1038/srep46307 (PMC5387733; doi:10.1038/srep46307)
Supplement: Supplementary Material [file srep46307-s1.doc]

**Upregulation of sestrins protect atriums** **against** **oxidative damage and fibrosis in human and** **experimental atrial fibrillation**

Zengxiang Dong^1^,*, Chaolan Lin^1^,*, Yujiao Liu^2^*, Hongbo Jin^3^*, Hong Wu^1^, Zhenjun Li^1^, Liping Sun^4^, Lu Zhang^4^, Xi Hu^1^, Yingying Wei^1^, Chengcheng Wang^1^ & Wei Han^1^

^1^ Department of Cardiology, The First Affiliated Hospital of Harbin Medical University, Harbin, China

^2^ Intensive Care Unit, Sir Run Run Shaw Hospital, School of Medicine, Zhejiang University, Zhejiang, China

^3^ Laboratory of Physiology, Harbin Medical University, Harbin, China

^4^ Department of Cardiology, The Second Affiliated Hospital of Harbin Medical University, Harbin, China

*These authors contributed equally to this work. Correspondence and requests for materials should be addressed to W.H. ( email: hanwei2 @medmail.com.cn)

**Supplementary material**

Table 1 Primers sequences used to obtain the full length of Sesns cDNA

|  | Sequence | | Product size（bp） |
| --- | --- | --- | --- |
| Sesn1 | sense | 5'-AAGGTACCATGCGCCTGGCCGCCGCGTC-3' | 1479 |
|  | antisense | 5'-ATGAATTCTCAGGTCATATACCGGGTAATG-3' |  |
| Sesn2 | sense | 5'-AAGGTACCATGATCGTAGCGGACTCCGAG-3' | 1443 |
|  | antisense | 5'-ATGAATTCTCAGGTCATGTAGCGGGTGATG-3' |  |
| Sesn3 | sense | 5'-AAGGTACCATGAACCGCGGTGGCAGCAG-3' | 1479 |
|  | antisense | 5'-ATGAATTCTCAGGTCAGATGCCGAGTTATG-3' |  |

Table 2 Short interfering RNAs targeting Sesns

| Targeting gene | Sequence |
| --- | --- |
| Sesn1 | 5'-CGGGCUAUCUGGAACUAUAdTdT-3' |
| Sesn2 | 5'-GACCAUGGCUACUCGCUGAdTdT-3' |
| Sens3 | 5'-GGAGCCUGAAGGUUUACAUdTdT-3' |
| Negtive Control | 5'-UUCUCCGAACGUGUCACGUdTdT-3' |

Table 3 Primer sequences for amplification of Sesns and GAPDH

|  | primers | | Product size（bp） |
| --- | --- | --- | --- |
| Sesn1 | sense | 5'-TCAGAATCCCTCGGCCACTA-3' | 264 |
|  | antisense | 5'-CTGCCGCCATGATTCCAATG-3' |  |
| Sesn2 | sense | 5'-TAGCCTGCAGCCTCACCTAT-3' | 109 |
|  | antisense | 5'-TATCTGATGCCAAAGACGCA-3' |  |
| Sesn3 | sense | 5'-CCAAGCAAATACGGCGGATG-3' | 137 |
|  | antisense | 5'-TGTAGAACTGGCTCCGCAAG-3' |  |
| GAPDH | sense | 5'-CCTTCCGTGTTCCTACCC-3' | 150 |
|  | antisense | 5'-CAACCTGGTCCTCAGTGTAG-3' |  |
